# A novel individualized strategy for cryoballoon catheter ablation in patients with paroxysmal atrial fibrillation

**DOI:** 10.1186/s12872-019-01295-1

**Published:** 2019-12-17

**Authors:** Jun Ding, Jing Xu, Wei Ma, Bingwei Chen, Peigen Yang, Yu Qi, Shan Sun, Aijuan Cheng

**Affiliations:** 1 Department of Cardiology, Tianjin Chest Hospital, Tianjin, People’s Republic of China, 261 South Taierzhuang Road, Jinnan District, Tianjin, 300200 China; 2 Tianjin medical university, Tianjin, People’s Republic of China

**Keywords:** Atrial fibrillation, Cryoablation, Catheter ablation, Cryoballoon, Dosing

## Abstract

**Background:**

The optimal dosage for cryoablation of atrial fibrillation (Cryo-AF) is still unknown. To evaluate the efficacy of an individualized freeze duration, we compared the clinical outcome of patients treated with a time-to-pulmonary vein isolation (TT-PVI) or temperature-guided ablation protocol to the outcome of patients treated with a conventional ablation protocol.

**Methods:**

A total of 164 consecutive patients were included in the study. One method employed was a conventional dosing protocol (at least 2 applications of 180 s each) (the Cryo-AF_Conventional_ group *n* = 84), and the second method had a shorter protocol that was based on the TT-PVI or achievement of − 40 °C within 60 s (the Cryo-AF_Dosing_ group *n* = 80)

**Results:**

We treated 656 pulmonary veins (PVs) with 1420 cryotherapy applications. The mean number of applications per patient was 8.7 ± 0.8, with no difference between groups (Cryo-AF_Conventional_, 8.7 ± 0.8 versus Cryo-AF_Dosing_,8.6 ± 0.8; *P* = 0.359). The Cryo-AF_Dosing_ group required significantly less total cryotherapy application time (990.60 ± 137.77versus 1501.58 ± 89.60 s; *P* < 0.001) and left atrial dwell time (69.91 ± 6.91 versus 86.48 ± 7.03 min; *P* < 0.001) than the Cryo-AF_Conventional_ group. Additionally, the Cryo-AF_Dosing_ group required significantly less total procedure time (95.03 ± 6.50 versus 112.43 ± 7.11 min; *P* < 0.001). We observed acute ATP-induced or spontaneous vein electric reconnections in 13 veins (1.98%) after 20 min. The reconnection rates between the Cryo-AF_Conventional_ and Cryo-AF_Dosing_ groups were similar in that 2.98 and 0.94% of the initially isolated veins were reconnected, respectively, (*P* = 0.061). There was no difference in the recurrence rate of free atrial arrhythmia after a 1-year follow-up, which were 78.75% in the Cryo-AF_Dosing_ group versus 78.57% in the Cryo-AF_Conventional_ group (*P* = 0.978).

**Conclusion:**

A novel Cryo-AF dosing protocol guided by temperature or the TT-PVI can be used to individualize an ablation strategy. This new protocol can lead to a significant reduction in duration of the procedure, the cryoenergy dosage and the left atrial dwell time. The procedure had equal safety and similar acute and 1-year follow-up outcomes compared to the conventional approach.

## Background

Atrial fibrillation (AF) is a very universal sustained cardiac arrhythmia. Pulmonary vein (PV) isolation (PVI) is a critical component of AF ablation. Cryoballoon (CB) ablation is highly effective in achieving PV isolation to treat AF [1–3].

The second-generation CB (CB2, Arctic FrontAdvance™, Medtronic, Inc.) was released with technical developments that allowed a larger and more consistent zone of freezing on the balloon surface. These developments translated into critical improvements in clinical and procedural outcomes when compared to the previous generation [4, 5]. However, as with all AF catheter ablation strategies, recurrence of arrhythmia and reconduction can arise throughout gaps when ablative lesions are incomplete, and conversely, complications can arise when collateral energy is transferred to nearby tissue. To prevent damage to extra cardiac structures, shorter times of application have been suggested. Currently used ablation strategies are often based on a fixed freeze-cycle duration of 180 s that is followed by a bonus freeze cycle of the same length after successful PVI. Currently, the ideal cryoablation of atrial fibrillation (Cryo-AF) dose is not well established, and predictors of durable electrical isolation are poorly known. Some clinical studies evaluated shorter freeze-cycle durations and determined whether the bonus application was actually necessary [6–9]. Prior research has suggested the time-to-pulmonary vein isolation (TT-PVI) procedure was a critical variable [7, 9].

This study evaluated the safety and efficacy of a shorter application strategy, based on the TT-PVI or achievement at − 40 °C within 60 s (Cryo-AF_Dosing_), compared with the conventional strategy (Cryo-AF_Conventional_).

## Methods

### Patients

This study was a single-centre, nonrandomized, open-label prospective clinical trial conducted at the Tianjin Chest Hospital. Patients with paroxysmal AF who received first-time CB ablation at the Tianjin Chest Hospital between October 2016 and January 2018 were enrolled in the trial. A total of 164 consecutive patients were included in the study. One method was a conventional dosing protocol. With this method, a 180-s freeze cycle was followed by a 180-s bonus freeze after acute PV isolation in the Cryo-AF_Conventional_ group. The second method had a shorter protocol that was based on the TT-PVI or achievement of − 40 °C within 60 s. Written informed consent was obtained from all patients. The inclusion criteria were age of 18 to 75 years, at least 2 paroxysmal AF episodes documented by electrocardiography, and no response to at least 1 antiarrhythmic drug. The exclusion criteria were acute coronary syndrome, persistent AF, left atrial size > 50 mm, intracardiac thrombi, uncontrolled heart failure, and a history of a previous AF ablation procedure. Patients with a left common trunk or right middle PV were also excluded from the analysis. Three experienced operators performed the procedures. The same individual operators carried out both ablation strategies. The Institutional Review Board of Tianjin Chest Hospital approved the study protocol. The clinical baseline characteristics of the study patients are shown in Table 1.

**Table 1:** Baseline characteristics of the patients

Characteristic	Cryo-AF_Dosing_(*n* = 80)	Cryo-AF_Conventional_(*n* = 84)	*P*
Age (y)	61 ± 7	59 ± 11	0.110
MaleGender	45 (56%)	48 (57%)	0.908
BMI (kg/m^2^)	25.8 ± 3.1	26.1 ± 3.5	0.557
Hypertension	43 (54%)	42 (50%)	0.631
Diabetes Mellitus	12 (15%)	13 (15%)	0.932
Stroke/TIA	7 (9%)	13 (15%)	0.188
CAD	23 (29%)	20 (24%)	0.472
CHA_2_DS_2_-VASc Score	1.7 ± 1.4	1.7 ± 1.4	0.809
LA Diameter (mm)	38.0 ± 3.4	38.8 ± 4.6	0.222
LVEF (%)	61.8 ± 4.3	60.8 ± 4.5	0.167

Values are given as mean ± SD or n (%), unless otherwise indicated. There are no different characteristics between both groups. *BMI* Body mass index, *CAD* Coronary artery disease, *CHA*_*2*_*DS*_*2*_*-VASc* Score for AF and stroke risk, *LA* Left atrial, *LVEF* Left ventricular ejection fraction, *TIA* Transient ischemic attack

### Ablation procedure

Conscious sedation was established before the CB ablation procedure was performed. Vital parameters (oxygen saturation, blood pressure, pulse) were continuously monitored. During the ablation procedure, it was recommended that active clotting times be maintained between 300 and 350 s by intravenous administration of heparin. We used biplane fluoroscopy with 30° right and 45° left anterior oblique views. The electrophysiological study and cryoablation procedure were performed. A diagnostic catheter was delivered via the right internal jugular vein and positioned within the coronary sinus. An exchange wire was placed in the left superior PV after a single transseptal puncture access was made with an SL-1 sheath (St. Jude Medical, Inc.) using the modified Brockenbrough method (BRK-1, St. Jude Medical, Inc.). The PV anatomy and left atrial was visualized by using PV angiography. The SL-1 sheath was then replaced with a steerable sheath (FlexCath Advance™, Medtronic, Inc.). A 20-mm circular mapping catheter (Achieve™, Medtronic, Inc.) was used to guide the CB2 inside the left atrium (LA) and to try real-time recordings from the focused PV in all patients. Once a PV was catheterized, the CB was inflated and advanced to the vein ostium, and PV occlusion was documented by injection of contrast medium. Our standard set of lesions included the left superior pulmonary vein (LSPV), which was dealt with first, followed by the left inferior PV (LIPV), right inferior PV (RIPV), and right superior PV (RSPV). The Achieve catheter was placed and used during cryoablation; the TT-PVI was recorded “online” when the PV potentials have been dissociated from left atrial activity or completely disappeared. While ablating near the RSPV or RIPV, the phrenic nerve (PN) was stimulated with a bipolar catheter placed in the superior vena cava, and diaphragmatic stimulation was realized by pacing the ipsilateral phrenic nerve with a 1500-ms cycle and a 10-mA cycle. The operator periodically noticed the diaphragmatic contraction by using fluoroscopy and placed a hand on the abdomen of the patient to evaluate the strength of contraction. Cryoablation was terminated immediately if loss or reduction of the pacing capture was detected. The balloon was repositioned at the PV antrum if the PN function recovered and ablation was then reattempted. Radiofrequency (RF) ablation was used to complete PV isolation if PN function failed to recover within 10–15 min.

**Fig. 1:**
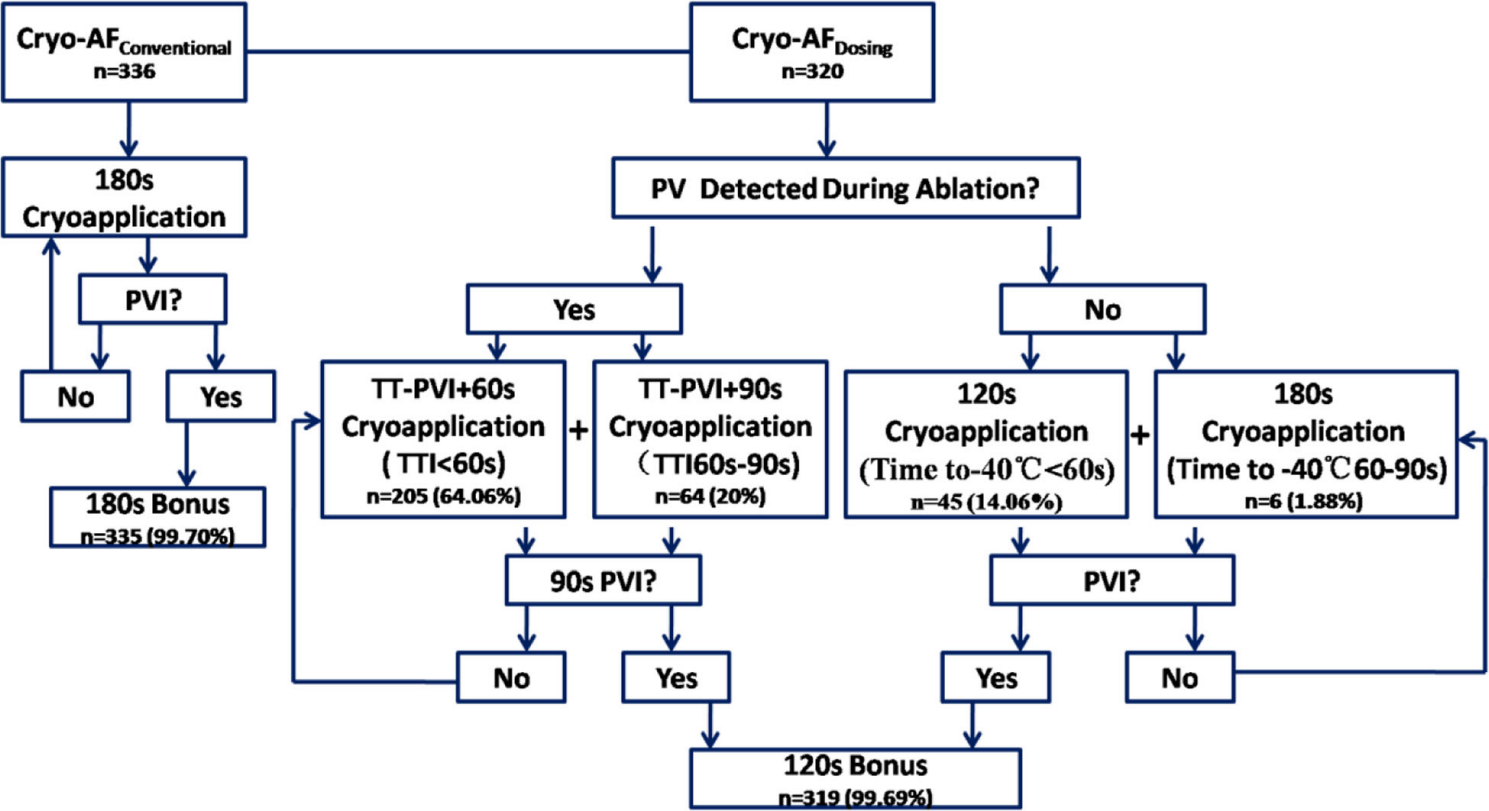
Protocol flowchart depicting cryoenergy dosing performed according to the TT-PVI or balloon temperature (Cryo-AF_Dosing_ group) or conducted with a conventional ablation protocol (Cryo-AF_Conventional_ group). In the Cryo-AF_Conventional_ group, cryoablation of 180 s was applieduntil bidirectional block of the vein was demonstrated. Following successful PVI, an additional 180-s freeze cycle was applied. In the Cryo-AF_Dosing_ group, the freeze duration was modified depending on the observed TT-PVI or balloon temperature. If a spontaneous TT-PVI was detectable, cryoenergy applications lasted for the TT-PVI plus 60–90 s. If the TT-PVI was < 60 s, a TT-PVI plus 60 s freeze cycle was applied. If the TT-PVI was between 60 and 90 s, a TT-PVI plus 90 s freeze cycle was applied. Additionally,if the TT-PVI was unavailable, according to the time from the beginning of ablation to the balloon temperature achievement of − 40 °C, cryoenergy of 120–180 s was applied until the vein was blocked. In other words, if the temperature reached − 40 °C within 60 s, a 120-s freeze cycle was applied, and if the temperature reached − 40 °C within 60–90 s, a 180-s freeze cycle was applied. In both cases, a 120-s bonus application was immediately administered. Cryo-AF = cryoablation of atrial fibrillation; PV = pulmonary vein; TT-PVI = time-to-pulmonary vein isolation; Time to − 40 °C, defined as the balloon freezing time from the beginning of the application to the moment of achievement of − 40 °C; 90S PVI, defined as pulmonary vein isolation in 90 s

In our study, we compared two cryotherapy application protocols. Cryoablation of 180 s was applied in the Cryo-AF_Conventional_ group until a bidirectional block of the vein was observed. Following successful PVI, an additional 180-s freeze cycle was applied to each PV. In the Cryo-AF_Dosing_ group, the freeze duration was modified relying the observed TT-PVI or balloon temperature. If a spontaneous TT-PVI was observed, cryoenergy applications lasted for the TT-PVI plus 60–90 s. If the TT-PVI was < 60 s, a TT-PVI plus 60-s freeze cycle was applied. If the TT-PVI was between 60 and 90 s, a TT-PVI plus 90 s freeze cycle was applied. We abandoned the cryoenergy applications that did not achieve TT-PVI within 90 s. The CB was placed in an alternate position to achieve a suitable TT-PVI. Additionally, if the TT-PVI was unavailable, cryoenergy of 120–180 s was applied until the vein was blocked according to the time from the beginning of ablation to the balloon temperature achievement of − 40 °C. In other words, if the temperature reached − 40 °C within 60 s, a 120-s freeze cycle was applied, and if the temperature reached − 40 °C within 60–90 s, a 180-s freeze cycle was carried out. A 120-s bonus operation was administered immediately in both cases, (Fig. 1). If the balloon temperature fell beneath − 55 °C, the cryoapplication was terminated, and the balloon was placed in a different position at the PV antra to avoid ultra-cold temperatures. The endpoint of cryoablation for PVs was isolation. This was demonstrated by pacing the stimulation of the LA as well as the corresponding PV. The nadir cryotemperature and total cryotherapy time per vein during every cryoablation were recorded, as was the total procedure time. The main adverse events related to cryoablation, specifically, pericardial effusion or tamponade, transient ischaemic attack (TIA) or stroke, PN palsy, atrial-oesophageal fistulas, and symptomatic PV stenosis, were also recorded. Isolation of all PVs was reassessed by documentation of entrance- and exit-block after a 20-min waiting period and in almost all cases following the administration of intravenous adenosine triphosphate (ATP). If PV reconnection was recorded, cryoenergy application of the reconnected PV was repeated.

### Cryokinetics

Three parameters of cryokinetics were introduced: TT-PVI, defined as the time from the beginning of the application to the moment the vein is blocked; time to − 40 °C, defined as the balloon freezing time from the beginning of the application to the moment of achievement of − 40 °C; and achievement − 40 °C within 60 s, which was defined as the balloon freezing time; that is, from the beginning of the application to the achievement of − 40 °C within 60 s.

### Postprocedural management and follow-up

Patients were discharged from the hospital within 3 days of the procedure. Oral anticoagulation treatment was initiated on the evening of ablation and was continued for at least 2 months. Antiarrhythmic drug (AAD) treatment was employed for 6 weeks and discontinued if the patient was free of AF recurrence. The patients were scheduled for follow-up visits at 1 and 3 months and then every 3 months after discharge from the hospital. The study considered a three-month blank period, and 12-lead electrocardiography and 24-h Holter recordings were acquired at each follow-up visit. Any symptoms after ablation were deemed eligible for Holter monitoring. Furthermore, telephone calls were also executed throughout the follow-up. If atrial tachycardia (AT), AF, or atrial flutter (AFL) lasted more than 30 s, cryoablation was said to have failed, and the failure date was recorded as the date of recurrence. Otherwise, the treatment was regarded as successful.

### Statistical analysis

Variables that composed a normal distribution were reported as the mean ± standard deviation and variables were compared with a Student’s t-test. The classification data were compared with the χ^2^ test or Fisher exact test. Kaplan-Meier method was used to predict the recurrence-free survival of AF, and the survival curve was compared with the log-rank test. The reported *P* values were calculated using a two-tailed test, and statistical significance was defined as *P* < 0.05. All statistical analyses were performed using IBM SPSS statistical software (Version 19.0, SPSS).

## Results

### Study population and procedural data

Data from 164 consecutive patients who were treated with Cryo-AF for the first time were evaluated. There were 80 patients who received Cryo-AF_Dosing_ and 84 patients who received Cryo-AF_Conventional_. Table 1 shows the baseline data for the study population. There were no differences in the clinical characteristics between the two groups. All baseline characteristics were similar.

### Acute procedural results

We treated 656 PVs with 1420 cryotherapy applications. During ablation, the potentials of PV in 83.23% of PVs could be monitored, with an average TT-PVI was 45.71 ± 21.26 s. In the Cryo-AF_Dosing_ group, 320 PVs were treated. Of these 320 PVs, 269 (84.06%) showed potentials during ablation and were treated with a cryotherapy dosage based on the TT-PVI. The other 51 (15.94%) PV potentials were not visible. Specifically, of 51 PVs, 45 (14.06%) PVs were treated with a cryotherapy dosage based on the achievement of − 40 °C within 60 s, and 6 (1.88%) PVs were treated with a cryotherapy dosage based on the achievement of − 40 °C within 60–90 s. Table 2 shows the procedure characteristics. Almost all (99.70%) veins were successfully isolated. Only 1 left superior PV in the Cryo-AF_Dosing_ group and 1 right inferior PV in the Cryo-AF_Conventional_ group remained reconnected after multiple attempts to isolation at the end of the procedure. The average number of applications per patient was 8.7 ± 0.8, there was no difference between the groups (Cryo-AF_Dosing_,8.6 ± 0.8 versus Cryo-AF_Conventional_, 8.7 ± 0.8; *P* = 0.359). The Cryo-AF_Dosing_ group required significantly less total cryotherapy application time (990.60 ± 137.77 versus 1501.58 ± 89.60 s; *P* < 0.001) and left atrial dwell time (69.91 ± 6.91 versus 86.48 ± 7.03 min; *P* < 0.001) than the Cryo-AF_Conventional_ group. Additionally, the Cryo-AF_Dosing_ group required significantly less total procedure time (95.03 ± 6.50 versus 112.43 ± 7.11 min; *P* < 0.001) than the Cryo-AF_Conventional_ group. The average radiography exposure time was 11.57 ± 0.88 min and there was no difference between the two groups. The requirement for RF ablation did not differ between the two groups.

**Table 2:** Procedural Characteristics

	Cryo-AF_Dosing_(*n* = 80)	Cryo-AF_Conventional_(*n* = 84)	*P*
PV isolated, n (%)^a^	319 (99.69%)	335 (99.70%)	0.972
RF ablation, n (%)^a^	1 (0.31%)	1 (0.30%)	0.972
PV potentials, n (%)^a^	269 (84.06%)	277 (82.44%)	0.578
PV acutely reconnected, n (%)^a^	3 (0.94%)	10 (2.98%)	0.061
Total applications per patient, n	8.6 ± 0.8	8.7 ± 0.8	0.359
Total cryotherapy time per patient,s	990.60 ± 137.77	1501.58 ± 89.60	< 0.001
LA dwell time, min	69.91 ± 6.91	86.48 ± 7.03	< 0.001
Total procedure time, min	95.03 ± 6.50	112.43 ± 7.11	0.000
Radiography exposure time,min	11.49 ± 0.85	11.65 ± 0.91	0.248

Values are given as mean ± SD or n (%), unless otherwise indicated

*Cryo-AF* Cryoablation of atrial fibrillation, *LA* Left atrial, *PV* Pulmonary vein, *RF* Radiofrequency

^a^PV; Cryo-AF_Dosing_(*n* = 320);Cryo-AF_Conventional_(*n* = 336)

We observed acute ATP-induced or spontaneous vein electric reconnections in 13 veins (1.98%) after 20 min. The reconnection rates between the Cryo-AF_Conventional_ and Cryo-AF_Dosing_ groups were similar in that 2.98 and 0.94% of the initially isolated veins were reconnected, respectively, (*P* = 0.061). In the Cryo-AF_Dosing_ groups, the reconnection rates between Cryo-AF_TT-PVI_ (2/269, 0.74%) and Cryo-AF_Time to − 40°C_ (1/51, 1.96%) were similar (*P* = 0.972). After extra applications, all veins were successfully reisolated.

The results from the procedure (analysed per vein) are summarized in Table 3. The PV potentials monitored during the procedure were not different between the two groups. The average number of applications and the average TT-PVI were also not different between the two groups. The total cryotherapy times per vein in the Cryo-AF_Dosing_ group, however, were significantly shorter than in the Cryo-AF_Conventional_ group (LSPV: 244.5 ± 41.6 s versus 368.0 ± 34.0 s, *P* < 0.001; LIPV: 244.0 ± 45.5 s versus 375.0 ± 45.0 s, *P* < 0.001; RIPV: 248.1 ± 40.3 s versus 392.0 ± 60.8 s, *P* < 0.001; RSPV: 236.8 ± 41.9 s versus 366.7 ± 21.2 s, *P* < 0.001, respectively). The average nadir temperatures in the right superior vein and left inferior vein were alike between the two groups. The nadir temperatures in the right inferior vein and left superior veins of the Cryo-AF_Dosing_ group, however, were lower than in the Cryo-AF_Conventional_ group (LSPV: -50.4 °C ± 3.7 °C versus − 48.4 °C ± 5.6 °C, *P* = 0.007; RIPV: -49.7 °C ± 4.8 °C versus − 46.8 °C ± 5.9 °C, *P* = 0.001).

**Table 3:** Acute Results of Cryoablation Procedure

Vein	Cryo-AF_Dosing_(*n* = 80)	Cryo-AF_Conventional_(*n* = 84)	*P*
Vein (LSPV)			
PV potentials, n (%)	77 (96.25%)	79 (94.05%)	0.513
TT-PVI, s	49.0 ± 20.7	52.9 ± 22.5	0.263
Nadir temperature,°C	-50.4 ± 3.7	−48.4 ± 5.6	0.007
Total cryotherapy time per vein, s	244.5 ± 41.6	368.0 ± 34.0	< 0.001
2 lesions per vein, n (%)	73 (91.25%)	73 (86.90%)	0.374
Late/ATP reconnection,n (%)	1 (1.25%)	2 (2.38%)	0.585
Vein (LIPV)			
PV potentials,n(%)	69 (86.25%)	70 (82.35%)	0.603
TT-PVI, s	45.2 ± 21.7	44.7 ± 23.2	0.124
Nadir temperature,°C	−45.5 ± 3.8	−44.2 ± 4.5	0.057
Total cryotherapy time per vein, s	244.0 ± 45.5	375.0 ± 45.0	< 0.001
2 lesions per vein, n (%)	70 (87.50%)	71 (84.52%)	0.583
Late/ATP reconnection,n (%)	1 (1.25%)	3 (3.57%)	0.648
Vein (RSPV)			
PV potentials, n (%)	66 (82.50%)	68 (80.95%)	0.798
TT-PVI, s	39.7 ± 18.9	40.2 ± 16.9	0.862
Nadir temperature,°C	−52.8 ± 4.4	−51.6 ± 3.8	0.061
Total cryotherapy time per vein, s	236.8 ± 41.9	366.7 ± 21.2	< 0.001
2 lesions per vein, n (%)	70 (87.50%)	75 (89.29%)	0.721
Late/ATP reconnection,n (%)	0	2 (2.38%)	0.497
Vein (RIPV)			
PV potentials, n (%)	57 (71.25%)	60 (71.43%)	0.980
TT-PVI, s	45.4 ± 23.6	46.8 ± 19.2	0.722
Nadir temperature,°C	−49.7 ± 4.8	−46.8 ± 5.9	0.001
Total cryotherapy time per vein, s	248.1 ± 40.3	392.0 ± 60.8	< 0.001
2 lesions per vein, n (%)	62 (77.50%)	60 (71.43%)	0.373
Late/ATP reconnection,n (%)	1 (1.25%)	3 (3.57%)	0.648

Values are given as mean ± SD or n (%), unless otherwise indicated. *LIPV* Left inferior pulmonary vein, *LSPV* Left superior pulmonary vein, *PV* Pulmonary vein, *RIPV* Right inferior pulmonary vein, *RSPV* Right superior pulmonary vein, *TT-PVI* Time to pulmonary vein isolation

### Complications

Complications, such as PN palsy, pericardial effusion, and groin vascular complications, were similar in two groups. In total, complications occurred in 4 of 80 (5%) patients in the Cryo-AF_Dosing_ group and 6 of 84 (7.14%) patients in the Cryo-AF_Conventional_ group (*P* = 0.566). There were no incidences of stroke, atrio-oesophageal fistula, cardiac tamponade, or death during follow-up (Table 4).

**Table 4:** Major complications

Adverse events	Cryo-AF_Dosing_(*n* = 80)	Cryo-AF_Conventional_(*n* = 84)	*P*
All, n (%)	4 (5%)	6 (7.14%)	0.566
Groin vascular complications, n (%)	3 (3.75%)	4 (4.76%)	0.749
Pericardial effusion, n (%)	0	1 (1.19%)	0.328
Phrenic nerve palsy, n (%)	1 (1.25%)	1 (1.19%)	0.972

Values are given as n (%)

### Follow-up

**Fig. 2:**
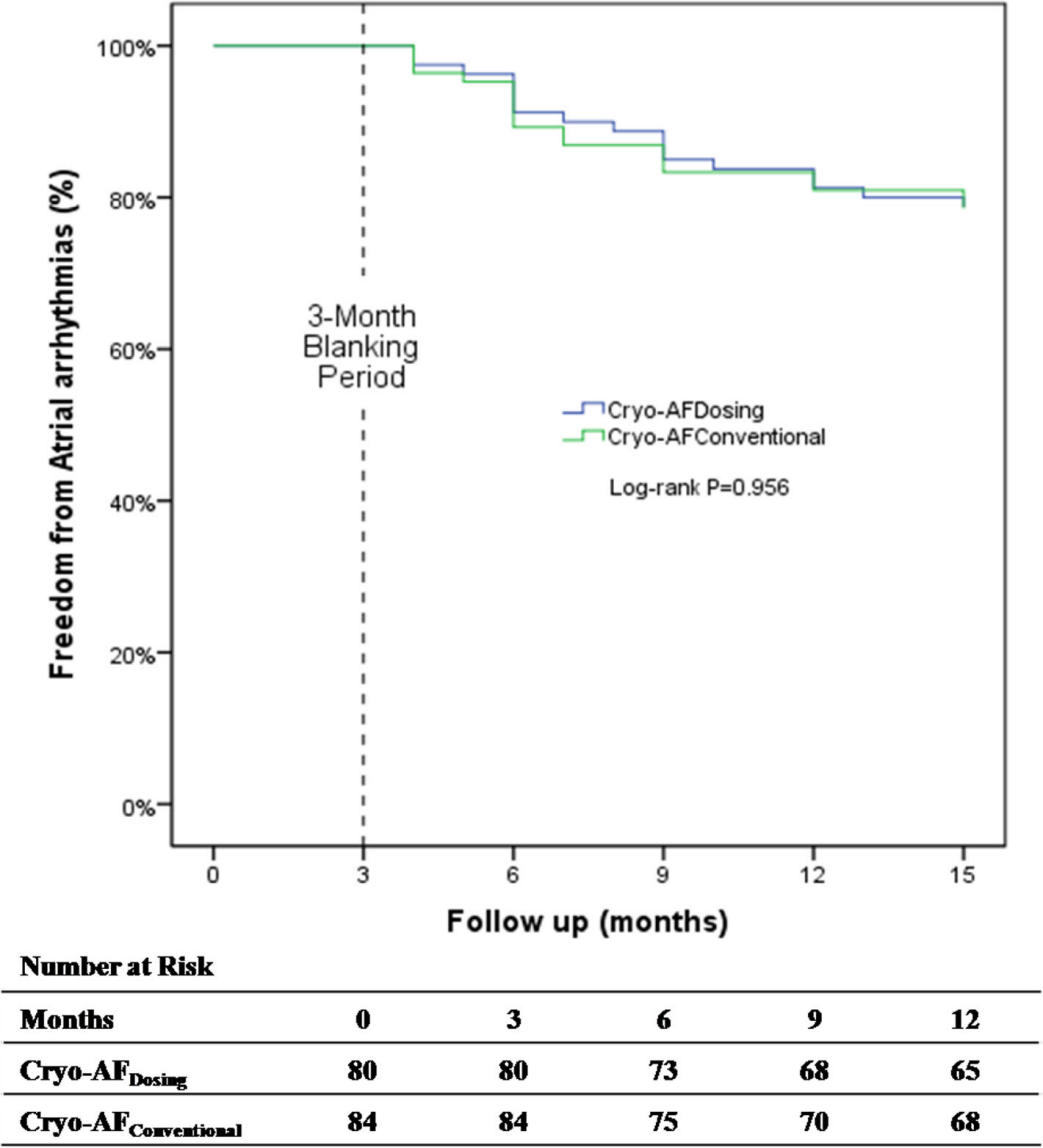
Kaplan-Meier curves indicating freedom from atrial arrhythmia recurrence during follow-up after cryoballoon pulmonary vein isolation with a Cryo-AF_Dosing_ group or a Cryo-AF_Conventional_ group

The 164 patients assigned to intervention were monitored with a 24-h Holter continuous electrocardiogram recording for each follow-up visit. There was no difference in the recurrence rate of free atrial arrhythmia without AADs between the two groups after three 3-month blanking period and after a 1-year follow-up. In addition, there were no significant differences between the Cryo-AF_Dosing_ and Cryo-AF_Conventional_ groups in freedom from recurrent atrial arrhythmias without AADs at 3, 6, 9, or 12 months (Table 5). Moreover, the Kaplan-Meier estimated freedom from atrial arrhythmias after the procedure did not differ significantly between the 2 groups (log-rank *P* = 0.956). Kaplan-Meier curves indicating freedom from atrial arrhythmias are shown in Fig. 2.

**Table 5:** Comparison of freedom from recurrent atrial arrhythmias between the two groups

	Cryo-AF_Dosing_(*n* = 80)	Cryo-AF_Conventional_(*n* = 84)	*P*
At 3 months	73 (91.25%)	75 (89.29%)	0.672
At 6 months	68 (85.00%)	70 (83.33%)	0.770
At 9 months	65 (81.25%)	68 (80.95%)	0.961
At 12 months	63 (78.75%)	66 (78.57%)	0.978

Values are given as n (%).*Cryo-AF* Cryoablation of atrial fibrillation

## Discussion

To the best of our knowledge, this was the first study that focused on a novel strategy for CB catheter ablation in patients with paroxysmal AF. This new strategy was based on a multiparametric evaluation (TT-PVI or achievement of − 40 °C within 60 s). In previous studies, according to the TT-PVI single parameter, 14.1–40% of the pulmonary vein potentials were unable to be recorded, and individualized cryoablation was not available [7, 9, 10]. In this study, with combined consideration of the balloon temperature and TT-PVI, nearly 100% of the pulmonary veins were treated with an individualized cryoablation strategy. The main aim of our study was to establish a simple and concise procedure, as well as an effective cryoablation strategy that could be easily implemented and consistantly replicated during Cryo-AF with different operators.

This study was a nonrandomized, single-center, open-label prospective analysis of the acute and long-term outcomes of cryotherapy used in patients with symptomatic AF. The strategy used a novel ablation-dosing algorithm that was guided by the TT-PVI or temperature. The main findings of this study were: first, achievement of − 40 °C within 60 s and TT-PVI are important considerations for both the efficacy and efficiency of cryoablation. This result indicated that TT-PVI can be used to individualize the ablation dosing protocol and reduce the freezing interval during Cryo-AF. Second, compared with previous investigations, this study increased the biophysics of ablation (achievement of − 40 °C within 60 s) to further expand the application range of cryoablation dosing. PVs ablation in our study group was applied with a nearly 100% individualized cryotherapy-dosing protocol. Third, the temperature-guided dosing algorithm or TT-PVI increased the efficiency of cryotherapy, which allowed for a shorter cryotherapy time and reduced left atrial dwell and procedure times. However, the rates of freedom from recurrent AF during long-term follow-up and complications were the same between the two cryoablation strategies.

Various procedures and biophysical indicators that were previously reported, as well as predictors of acute and persistent PV isolation, were carefully evaluated during the development of the expected algorithm. Finally, based on available data, we developed the recommended dosing regimen, which depended on the TT-PVI or achievement of − 40 °C within 60 s.

The TT-PVI has been identified as a prognostic indicator for PVI durability in CB ablation. In this previous study, the TT-PVI dosing strategy was used to determine the minimal effective time required to create a transmural lesion. A recent study conducted on animal models concluded that the CB2 achieved sustained PVI and that the duration of the freezing cycle was significantly shortened. In the canine model, effective PVI was found in the shortest administration group (TT-PVI + 60 s), with an average total ablation time of approximately 90 seconds [11]. Several studies have evaluated whether the TT-PVI dosing strategy could reduce cryoapplications and quantify ablation energy. The Individualized Cryoballoon Energy Trial (ICE-T) documented comparable outcomes when the freezing time was reduced. In the ICE-T, 50 subjects were treated with a standard freezing protocol of 240 s and an additional freezing protocol of 240 s. The group was compared to 50 patients who were treated with the TT-PVI dosing strategy. A bonus application was administered only if TT-PVI was > 75 s. For 88% of PVs, a single-freeze protocol was used and TT-PVI was observed in 79% of PVs regardless of cohort designation. There were no differences in recurrence of AF during 1-year follow-up [12]. Aryana et al. showed the biophysical and procedural characteristics of 112 patients who underwent a secondary procedure after CB2 cryotherapy of AF and PVI in 60 s were predictive of continued success [13]. Ciconte et al. also suggested that TT-PVI be regarded as an independent predictor of atrial arrhythmia recurrences [8]. Ferrero-de-Loma-Osorio et al. performed a TT-PVI-guided dosing procedure in 69 patients compared with 68 control patients who underwent cryotherapy using a usual procedure. The authors found that the dosing procedure was associated with a shorter total ablation time (19.4 ± 4.3 versus 28.3 ± 7 min; *P* < 0.001), as well as left atrial dwell time (92 ± 23 versus 104 ± 25 min; *P* < 0.01) and total procedural time (85 ± 25 versus 97 ± 22 min; *P* < 0.01), than that of the conventional procedure. The overall incidence of adverse events and 12-month freedom from all atrial arrhythmia recurrence (78.3% vs 79.4%; *P* = 0.869) were similar [9].

When TT-PVI was not achievable, the balloon temperature could be used to guide ablation. Cryosurgical animal studies have demonstrated that maximal tissue damage occurs when a temperature below − 40 °C is obtained with a rapid temperature drop (> 10 °C/min) [14]. Recent articles have reported that achieving − 40 °C in 60 s is an independent predictor of durable PVI [15, 16]. Iacopino et al. compared 52 patients with temperature-assisted cryoablation to 52 patients with TT-PVI-guided cryoablation. The studies showed that a temperature-guided strategy that was based on achieving − 40 °C within 60 s without real-time recordings was successful in producing PVI. Acute PVI was obtained in 99% of the PVs for the temperature-assisted cryoablation cohort. There was no difference in recurrence at a mean follow-up of 12 months between the two groups (85% vs. 88%, respectively; *P* = 0.56) [15]. Giuseppe Ciconte’s research revealed that achievement of − 40 °C within the first minute could independently predict durable PVI^16^. In a study by Wei et al., achievement of − 40 °C within 60 s was observed in 67% of the PVs and there was no association with acute reconnections, which was consistent with previous investigations [17].

We designed a TT-PVI or temperature-based individualized protocol. In a conventional approach, some fixed cryoablation times may result in either insufficient or excessive durations. Prolonging the ablation over this duration did not result in further increases in the lesion depth or volume. Therefore, a longer than required ablation time may not only prolong the operation times but may also increase the risk of complications in important extracardiac structures, such as the esophagus, bronchial trees, or the PN. The use of a novel Cryo-AF individualized strategy may provide a more tailored approach that balances the effects of ablation while avoiding unnecessary over-ablation in thin-walled high-risk areas. There was a low incidence of severe events when this new procedure was used, and there was no difference between the two groups in the incidence of severe events. The TT-PVI or temperature-guided ablation protocol significantly reduced left atrial dwell, ablation, and procedure times. Our data could not deduce whether this finding translates into a reduction in adverse events, because overall adverse events were very low and the number of patients in this study was still too small to confidently estimate the incidence of complications. Further studies with a larger samples of patients are necessary in order to evaluate any potential reduction in complication rates. In contrast, insufficient freeze times may result in a higher recurrence rate of AF. In this study, a second segmental ablation was performed in PVs to ensure a wider range circumferential lesion to increase operational effectiveness. Future studies will focus on the clinical outcome of applying two different ablation strategies: bonus or nonbonus with application time based on the TT-PVI or temperature. We therefore strongly suggest that the duration of cryoapplications should be guided through a systematic approach that is tailored based on objective and quantifiable measures of cryoablation during Cryo-AF. A TT-PVI or temperature-based individualized protocol has the potential to enhance PVI results and deserves further study. Future studies could determine whether patient-specific factors can be used to derive truly individualized optimal cryoablation dosages for each patient.

### Limitations

The findings of this study are based on a single-centre experience and only recruit a limited number of patients in a non-random manner. We cannot rule out unknown confounding variables because the treatment assignment was nonrandomized. Randomized studies of a large number of patients may be required in the future to show the long-term effect of a TT-PVI or temperature-guided strategy.

## Conclusions

A novel Cryo-AF dosing protocol guided by temperature or the TT-PVI can be used to individualize an ablation strategy. This new protocol can lead to a significant reduction in duration of the procedure, the cryoenergy dosage and the left atrial dwell time. The procedure had equal safety and similar acute and 1-year follow-up outcomes compared to the conventional approach.

## Data Availability

The datasets generated and analysed during the current study are notpublicly available due to the Tianjin Chest Hospital regulations, but are available from the corresponding author on reasonable request.

## Abbreviations

AF: Atrial fibrillation
CB: Cryoballoon
CB2: Second-generation CB
Cryo-AF: Cryoablation of atrial fibrillation
PV: Pulmonary vein
PVI: Pulmonary vein isolation
RF: Radiofrequency
TT-PVI: Time-to-pulmonary vein isolation

## References

[1] Packer DL, Kowal RC, Wheelan KR (2013). Cryoballoon ablation of pulmonary veins for paroxysmal atrial fibrillation: first results of the north American Arctic front (STOP AF) pivotal trial. J Am Coll Cardiol.

[2] Kuck KH, Brugada J, Furnkranz A (2016). Cryoballoon or radiofrequency ablation for paroxysmal atrial fibrillation. N Engl J Med.

[3] Knight BP, Novak PG, Sangrigoli R (2019). Long-term outcomes after ablation for paroxysmal atrial fibrillation using the second-generation Cryoballoon: final results from STOP AF post-approval study. JACC Clin Electrophysiol.

[4] Furnkranz A, Bordignon S, Schmidt B (2013). Improved procedural efficacy of pulmonary vein isolation using the novel second-generation cryoballoon. J Cardiovasc Electrophysiol.

[5] Giovanni GD, Wauters K, Chierchia GB (2014). One-year follow-up after single procedure Cryoballoon ablation: a comparison between the first and second generation balloon. J Cardiovasc Electrophysiol.

[6] Miyamoto K, Doi A, Hasegawa K (2019). Multicenter study of the validity of additional freeze cycles for Cryoballoon ablation in patients with paroxysmal atrial fibrillation. Circ Arrhythm Electrophysiol.

[7] Aryana A, Kenigsberg DN, Kowalski M (2017). Verification of a novel atrial fibrillation cryoablation dosing algorithm guided by time-to-pulmonary vein isolation: results from the Cryo-DOSING study (Cryoballoon-ablation DOSING based on the assessment of time-to-effect and pulmonary vein isolation guidance). Heart Rhythm.

[8] Ciconte G, de Asmundis C, Sieira J (2015). Single 3-minute freeze for second-generation cryoballoon ablation: one-year follow-up after pulmonary vein isolation. Heart Rhythm.

[9] Ferrero-de-Loma-Osorio A, Garcia-Fernandez A, Castillo-Castillo J, et al. Time-to-Effect-Based Dosing Strategy for Cryoballoon Ablation in Patients With Paroxysmal Atrial Fibrillation: Results of the plusONE Multicenter Randomized Controlled Noninferiority Trial. Circ Arrhythm Electrophysiol. 2017;10(12):e005318. 10.1161/CIRCEP.117.005318 29247029

[10] Boveda S, Providencia R, Albenque JP (2014). Real-time assessment of pulmonary vein disconnection during cryoablation of atrial fibrillation: can it be 'achieved' in almost all cases?. Europace..

[11] Su W, Coulombe N, Kirchhof N, Grassl E, Wittenberger D (2018). Dosing of the second-generation cryoballoon using acute time-to-pulmonary vein isolation as an indicator of durable ablation in a canine model. J Interv Card Electrophysiol.

[12] Chun KR, Stich M, Furnkranz A (2017). Individualized cryoballoon energy pulmonary vein isolation guided by real-time pulmonary vein recordings, the randomized ICE-T trial. Heart Rhythm.

[13] Aryana A, Mugnai G, Singh SM (2016). Procedural and biophysical indicators of durable pulmonary vein isolation during cryoballoon ablation of atrial fibrillation. Heart Rhythm.

[14] Gage AA, Baust J (1998). Mechanisms of tissue injury in cryosurgery. Cryobiology..

[15] Iacopino S, Mugnai G, Takarada K (2017). Second-generation cryoballoon ablation without the use of real-time recordings: a novel strategy based on a temperature-guided approach to ablation. Heart Rhythm.

[16] Ciconte G, Mugnai G, Sieira J (2015). On the quest for the best freeze: predictors of late pulmonary vein reconnections after second-generation Cryoballoon ablation. Circ Arrhythm Electrophysiol.

[17] Wei HQ, Guo XG, Zhou GB (2018). Pulmonary vein isolation with real-time pulmonary vein potential recording using second-generation cryoballoon: procedural and biophysical predictors of acute pulmonary vein reconnection. Pacing Clin Electrophysiol.

